# Novel Cell Cycle Inhibitors Decrease Primary and Metastatic Breast Cancer Growth In Vivo

**DOI:** 10.3390/cancers18030466

**Published:** 2026-01-30

**Authors:** Mir Shahid Maqbool, Yongzhan Zhang, Karin Strittmatter, Ana Gvozdenovic, Simran Asawa, Masroor A. Paddar, Mukesh Kumar, Umed Singh, Parvinder Pal Singh, Nicola Aceto, Fayaz Malik

**Affiliations:** 1 Division of Cancer Pharmacology, CSIR-Indian Institute of Integrative Medicine, Sanat Nagar, Srinagar 190005, Jammu & Kashmir, India; mirshahid070@gmail.com (M.S.M.); mpaddar@salud.unm.edu (M.A.P.); 2 Department of Biology, Institute of Molecular Health Sciences, Swiss Federal Institute of Technology Zurich (ETH Zurich), 8093 Zurich, Switzerland; yongzhan.zhang@biol.ethz.ch (Y.Z.); karin.strittmatter@biol.ethz.ch (K.S.); ana.gvozdenovic@biol.ethz.ch (A.G.); simran.asawa@biol.ethz.ch (S.A.); 3 Division of Medicinal Chemistry, CSIR-Indian Institute of Integrative Medicine, Jammu 180001, Jammu & Kashmir, India; mukeshbhogal@gmail.com (M.K.); beniwal36@gmail.com (U.S.); ppsingh@iiim.res.in (P.P.S.)

**Keywords:** breast cancer, circulating tumor cells, drug screen, mouse model

## Abstract

**Background:** Breast cancer is one of the most frequently diagnosed cancers worldwide, with metastasis contributing to high mortality rates. Current treatments for metastatic disease are limited, emphasizing the urgent need for novel therapeutic approaches. **Methods**: We conducted a small-molecule drug screen utilizing patient-derived circulating tumor cells (CTCs) as a platform to identify potential anti-cancer agents. We used a dye combination and a high-content imaging microscope to evaluate cellular viability upon compound treatment. Among the 250 small molecules tested, potential hits were identified. The efficacy of these compounds was investigated using in vitro and in vivo studies in mouse breast cancer models. Bulk RNA sequencing of treated cancer cells was performed to identify differentially expressed genes, with Gene Ontology enrichment analyses conducted for their functional characterization. **Results**: Our screen of a 250 small-molecule library led to the identification of five hits, derivatives of meriolins known to display cyclin-dependent kinase (CDK-2/9) inhibitory activity. Subsequent in vitro and in vivo studies validated the efficacy of these compounds in inhibiting cell cycle, tumor growth, and consequently, metastatic colonization in mouse breast cancer models. Treatment with single agents (15 mg/kg) in breast cancer mouse models demonstrated good tolerability in vivo. Transcriptome profiling of treated cancer cells revealed alterations in pathways associated with cell cycle regulation, providing mechanistic insights into the anti-cancer effects of the compounds. **Conclusions**: By integrating drug screens, transcriptomic analysis, and in vivo validation, our study contributes to the identification of novel promising candidates for the treatment of breast cancer.

## Simple Summary

Breast cancer is a leading cancer worldwide, with metastasis causing high death rates. Currently, patients with overt metastatic disease are considered incurable. We used patient-derived circulating tumor cells (CTCs) to screen 250 small molecules, identifying five compounds with potential anti-cancer effects. These compounds, which inhibit cyclin-dependent kinase (CDK-2/9), effectively reduced tumor growth and metastasis in mouse breast cancer models. Transcriptome profiling of treated cancer cells revealed alterations in pathways associated with cell cycle regulation, providing mechanistic insights into the anti-cancer effects of the compounds. Altogether, these results contribute to providing new therapeutic tools that could be implemented for the treatment of breast cancer.

## 1. Introduction

Breast cancer is among the most commonly diagnosed cancers, accounting for 685,000 fatalities in 2020 worldwide [1]. Its high mortality rate is associated with the development of metastases, a complex process involving the spread of cancer cells from the primary tumor site to secondary organs. At present, treating patients with metastatic disease is challenging, and this still represents an unmet medical need [2].

Investigations of circulating tumor cells (CTCs) have significantly expanded our understanding of how cancer spreads, as these cells, which detach from growing cancerous lesions and enter the bloodstream, play a pioneering role in the metastatic process [3]. They circulate in the blood either as individual cells or as multicellular aggregates known as CTC clusters, with the latter being associated with elevated metastatic potential when compared to single CTCs [4,5]. Furthermore, the presence of CTC clusters in patients with various types of cancer is linked to poor prognosis [6]. Importantly, breast cancer CTCs, propagated as 3D ex vivo cultures under hypoxic conditions, retain both molecular and genetic features of the tumor of origin and of freshly isolated CTCs [7,8]. As clinically relevant models, CTC-derived models can serve as drug screen platforms that enable drug susceptibility testing and discovery of novel compounds with anti-cancer activity.

Small molecules, as a chemical tool, offer a unique opportunity to investigate cancer vulnerabilities, leading to the identification of new biological targets and potential new drug-like candidates. In this study, we screened 250 compounds from the small-molecule repository of the CSIR-Indian Institute of Integrative Medicine, including natural products and their new derivatives, for cytotoxic potential against CTC-derived cells. This chemical screen led to the identification of five hits of marine-based scaffolds, derivatives of meriolins (a hybrid of two marine natural products: variolins and meridianins). Variolins and meridianins represent the marine natural products belonging to pyridopyrrolo pyrimidine and 3-pyrimidinyl indole. Based on these scaffolds, earlier, our group designed and generated new derivatives showing promising CDK2 and CDK9 inhibitory activities [9]. Given recent FDA approvals of CDK inhibitors in breast cancer specifically targeting CDK4 and CDK6 either alone or in combination with other treatments, we decided to pursue further investigations. Interestingly, the identified five hits are derivatives of the same scaffold and possess strong CDK2 and CDK9 inhibitory activity. We further provide in vivo validation of the best candidates along with gene expression profiling to gain additional insights into their mechanisms of action.

## 2. Materials and Methods

### 2.1. Cell Culture

BR16 cells, originating from CTCs from a metastatic breast cancer patient [7], were cultured as previously described [10]. BR16 cells were grown as suspension cultures in RPMI medium (Invitrogen, Thermo Fisher Scientific, Waltham, MA, USA, 52400-025) supplemented with 1B27 (Invitrogen, 17504-044), 1% antimycotic/antibiotic, human recombinant Fibroblast Growth Factor (FGF; 20 ng/mL, GIBCO, Thermo Fisher Scientific, Waltham, MA, USA, 100-18B), and human recombinant Epidermal Growth Factor (EGF; 20 ng/mL; GIBCO, PHG0313) in a humidified incubator at 37 °C with 5% O_2_ and 5% CO_2_, using ultra-low attachment plates (Sarstedt, Nümbrecht, Germany, 83.3920.500). 4T1 murine breast cancer cells were obtained from ATCC (Manassas, VA, USA, CRL-2539) and grown in DMEM7F-12 high glucose (GIBCO, 11330-057) supplemented with 10% heat-inactivated FBS (GIBCO, 10500064) and 1% antimycotic/antibiotic (GIBCO, 15240-062) in a humidified incubator at 37 °C with 20% O_2_ and 5% CO_2_. 4T1 cells overexpressing Green Fluorescent Protein (GFP) and Luciferase (Luc) were generated as previously reported [11].

### 2.2. Chemistry

The identified hits were designed and synthesized as part of new-generation CDK inhibitors. The synthesis of three identified hits, namely IIIM-368, US-463, and US-464, were reported in [9]. The remaining two active hits (DF-06 and US-748) were synthesized by a similar strategy, and details are provided in the Supplementary Data (Scheme S1). Paclitaxel was purchased from (Alfa Aesar, Thermo Fisher Scientific, Ward Hill, MA, USA, J62734).

### 2.3. Small-Molecule Library Screen

A library containing 250 compounds was screened against the breast CTC line BR16. Each compound was resuspended using CTC medium at a 15 µM concentration. Forty µL of the medium, containing 8000–12,000 highly proliferative CTC-derived cells with >90% viability, was added to each well of a flat-bottom, clear, ultra-low-attachment 96-well plate (Corning, Corning, NY, USA 3474). To achieve a final concentration of 5 µM, 20 µL of 15 µM stock compound solution was added to the cells. The experiments were performed in duplicate. Plates were incubated in hypoxic conditions (5% oxygen) for 48 h, and then 40 µL was transferred to a 96-well black/clear tissue culture-treated plate (BD Falcon, Franklin Lakes, NJ, USA, 353219). Cells treated with DMSO (0.25%) alone were used as the control. Subsequently, 20 µL of D-PBS (Invitrogen, Cat#14190169) or dye mix (final concentrations of 4 µM Hoechst (Invitrogen, H21486) and 4 µM TOTO-3 (Invitrogen, Cat# T3604)) was added, and cells were stained for one hour at 37 °C. Plates were scanned using an Operetta High-Content Imaging System (Perkin Elmer, Waltham, MA, USA), and CTC cluster analysis was performed using Columbus Image Data Storage and Analysis System (Perkin Elmer).

### 2.4. Kinase Activity Assay

Kinase inhibitory activity measurements were carried out using biochemical assays, including radioactive assays and ADP-Glo luciferase assays. Detailed information regarding these assays can be found in our previously published work [9].

### 2.5. Cell Viability Assay

The potential inhibitory effect of identified compounds on cell proliferation was measured through a calorimetric assay using dye MTT (3-(4,5-dimethylthiazole-2-yl)-2,5 diphenyltetrazolium bromide), as reported earlier [12]. The mouse mammary carcinoma cell line 4T1 (2000 cells/well) was seeded in 96-well flat-bottom plates (Nunc, Thermo Fisher Scientific, Rochester, NY, USA). After 24 h of incubation, the cells were treated with given compounds at various concentrations for 48 h. Four hours before termination of the experiment, 20 µL of MTT dye (2.5 mg/mL) was added to each well. The experiment was terminated, the supernatant was discarded, and the formazan crystals were dissolved in DMSO (150 µL/well). Absorbance was measured using a spectrophotometer at 570 nm. Growth inhibition was calculated by comparing the absorbance of treated versus untreated cells.

### 2.6. Western Blotting

Prior to Western blotting, cells were left untreated or treated for 12 h with an increasing concentration of selected compounds. For immunoblotting, proteins were extracted from cells using RIPA lysis buffer, and protein estimation was performed using the Bradford assay. Equal amounts of protein from each sample were subjected to 8% SDS-PAGE and then transferred onto a polyvinylidene difluoride (PVDF) membrane for 120 min at 4 °C at 100 V. Then, the PVDF membrane was blocked with 5% non-fat milk or 5% BSA in order to avoid non-specific binding, followed by incubation with target primary antibodies (p-Rpb1 CTD (Ser2/5) (4735 CS), Rpb1 CTD (2629 CST), p-Rb (Ser807/811) (8516 CST), Rb (4H1) (mouse mAb 9309 CST), and β-actin A3854 (Sigma, Merck KGaA, St. Louis, MO, USA)) and their respective HRP-conjugated secondary antibodies. The membrane was incubated with chemiluminescence horseradish peroxidase (HRP) substrate, the signal was captured on the Chemidoc system, and quantification was performed using ImageJ software (2.16.0). The chemicals used were purchased from Sigma.

### 2.7. Orthotopic Breast Cancer Mouse Model

Female Balb/c mice (18–23 g) were procured from the animal facility of the institute with approval from the Institutional Animal Ethical Committee (IAEC no. 1361/73/8/21). Experiments were performed under the set guidelines from the Institutional Animal ethical Committee (IAEC), and the Committee for Control and Supervision of Experiments on Animals (CCSEA), Government of India. Mammary tumors were established by orthotopically injecting 1 × 10^6^ 4T1 cells in 100 µL of PBS/Matrigel mixture (1:1 ratio) into the mammary fat pads of the mice [12]. As tumors reached a volume of approximately 100 ± 10 mm^3^, the mice were randomly divided into five groups with 6 mice/group, and treatments were initiated. Group I served as the vehicle control, group II received paclitaxel (10 mg/kg), group III was given US-463 (15 mg/kg), group IV was given US-464 (15 mg/kg), and group V was given DF-06 (15 mg/kg) through the tail vein twice a week for three weeks. Tumor growth was measured twice weekly with vernier calipers, and the volume of the tumors was calculated using the formula (L × B^2^/2) mm^3^ (L indicates length; B indicates width). The experiments were repeated twice.

### 2.8. Experimental Metastasis Breast Cancer Mouse Model

The experimental metastasis study was conducted in strict compliance with both institutional and cantonal guidelines and in accordance with Swiss Animal Protection Law (approved license number ZH100/21). Immunocompromised NSG (NOD-scid-Il2rgnull) 8-week old male mice (23–27 g) were obtained from The Jackson Laboratory (Bar Harbor, ME, USA). The animals were anesthetized with isoflurane and administered 100,000 live 4T1-GFP-Luc cells resuspended in 100 µL of PBS (Invitrogen, 14190169) via the tail vein. Prior to injection, the cells were treated with individual compounds at their respective IC50 concentrations, each dissolved in DMSO, for a duration of 48 h. The specific IC_50_ values used were as follows: US-463 at 205.6 nM, US-464 at 276.1 nM, and DF-06 at 1585 nM. Control cells were treated with 0.25% DMSO. To determine the number of live cells, cell counting was then carried out using the Invitrogen Countess™ 3 FL Automated Cell Counter (Thermo Fisher Scientific, Waltham, MA, USA). Each experimental group consisted of 4 animals. The metastatic burden was determined via in vivo and ex vivo bioluminescence imaging using IVIS Lumina II system (PerkinElmer, Waltham, MA, USA). Animals were sacrificed 13 days after tumor cell injection. To evaluate Ki67 expression in a short-term study, the animals (*n* = 2) were euthanized 24 h following tumor cell injection, after which lungs were harvested for analysis.

### 2.9. Immunohistochemistry

Formalin-fixed, paraffin-embedded primary tumor sections were deparaffinized at 70 °C for 25 min, followed by washing in xylene and rehydrated in graded alcohols (ethanol). For antigen retrieval, tissues were fixed in ImmunoDNA retrieval with EDTA buffer (Bio SB, Santa Barbara, CA, USA, BSB 0030) for 30 min. After washing with washing buffer Immuno/DNA Washer (Bio SB, BSB 0150), endogenous peroxidase activity was quenched with 3% hydrogen peroxide for 20 min, followed by two 5 min washes in washing buffer. The specimens were then incubated with the primary antibody Ki67 (CST, Danvers, MA, USA, 12202S) for 2 h at 4 °C in a humid chamber, followed by two 5 min washes in washing buffer. Then secondary antibody conjugated with horseradish peroxidase (secondary antibody anti-rabbit, Thermo Fisher Scientific, Rochester, NY, USA, A9169-2ML) was added and incubated for 40 min at room temperature. The slides were stained with 3,3′-diaminobenzidine tetrahydrochloride (DAB, Bio SB, Santa Barbara, CA, USA, BSB 001) until a brown color started to develop. They were then washed twice with washing buffer for 5 min each, followed by counterstaining with hematoxylin. Afterward, the slides underwent dehydration in a series of ethanol solutions, cleared with xylene, and mounted using DPX mountant (Sigma-Aldrich, Merck KGaA, St. Louis, MO, USA). Observation and photography were performed using the EVOS M7000 imaging system (Invitrogen Thermo Fisher Scientific microscope), and grading of slides was performed blindly by a pathologist.

### 2.10. Immunofluorescence

Lung samples were fixed in 4% paraformaldehyde at 4 °C overnight, followed by incubation in 30% sucrose before embedding in OCT. Ten µm thick OCT sections were obtained for immunofluorescence staining protocol. The sections were permeabilized for 20 min with 0.1% Triton-X-100 in PBS and blocked in normal goat serum for 1 h at room temperature. Primary antibody staining was performed overnight at 4 °C using anti-GFP antibody (Antibodies Inc., Davis, CA, USA, GFP-1020) and anti-Ki67 antibody (Invitrogen, 14-5698-82), each at a 1:200 dilution. After washing with PBS, tissue sections were incubated for 1 h at room temperature with secondary antibodies—goat anti-chicken Alexa Fluor^TM^ 568 (Invitrogen, A-11041) and goat anti-rat DyLight^TM^ 755 (Invitrogen, SA5-10023)—each at 1:400 dilution. Nuclei were counterstained with DAPI (Sigma-Aldrich, Merck KGaA, St. Louis, MO, USA, D9542) at 1 µg/mL working concentration. Slides were mounted with Prolong^TM^ Gold Antifade mountant (Invitrogne, P36930) and left to air dry overnight before imaging using the Evident/Olympus VS200 slide scanner (Evident Corporation, Tokyo, Japan). All images were analyzed by Qupath (0.5.1) and ImageJ (2.16.0, NIH, Bethesda, MD, USA). Ki67 positivity was expressed as the percentage of Ki67-positive tumor nuclei among all tumor nuclei.

### 2.11. RNA Sequencing

Cells were treated with either a control vehicle (0.25% DMSO) or 5 µM of the above-mentioned compounds for two and six hours, respectively. Total RNA was extracted using the RNeasy Mini Kit (Qiagen, Hilden, Germany) as per the manufacturer’s instructions. Total RNA-seq reads were trimmed from the 3′ end until the final base had a quality score > 30, utilizing Trimmomatic version 0.33 [13], and aligned to the human reference genome UCSC hg38 using STAR (v2.7.9a) [14]. Aligned reads were counted using HTSeq (v2.0.9) [15]. Normalization of the data and differential gene expression analysis were performed using DESeq2 (1.50.2) [16] with default parameters. Significantly differentially expressed genes were identified using an adjusted *p*-value threshold of 0.01 and an absolute log2 fold change greater than 0.5. Subsequently, Gene Ontology (GO) term analysis was performed using Metascape (2.16.0) [17] to analyze the enrichment of biological processes and pathways among the significant genes.

### 2.12. Wound Healing Assay

Briefly, 4T1 cells (1 × 10^6^ cells per well) were seeded into 6-well plates and cultured for 48 h to achieve near confluence. A uniform wound was then created using a 10 μL pipette tip (Corning, #4808). Cell debris was removed, and the monolayer was gently washed once with 1 mL of growth medium to smooth the wound edges. Cells were then incubated in DMEM supplemented with 1% FBS and treated with DF-06 (792 nM), US463 (102 nM), or US464 (138 nM) for 16 h. Control wells received DMEM supplemented with 1% FBS and 0.25% DMSO. The initial wound area was recorded, and gap areas were subsequently measured at 16 h. Wound closure was analyzed with ImageJ (2.16.0, NIH, Bethesda, MD, USA).

### 2.13. Statistical Analysis

Data analysis, statistical tests, and visualization were conducted in R (version 2021.09.0 + 351; R Foundation for Statistical Computing, Vienna, Austria) and GraphPad Prism (v9.0).

## 3. Results

### 3.1. Identification of Compounds Through a Small-Molecule Library Screen in CTC-Derived Cells

**Figure 1:**
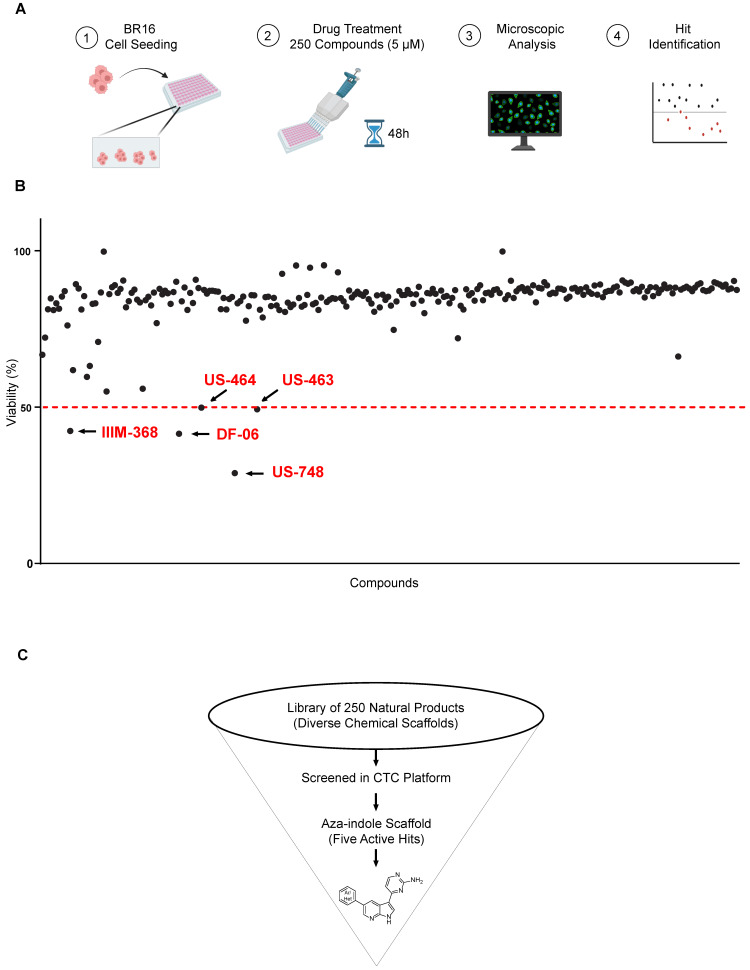
Screen for natural and synthetic compounds that reduce the viability of BR16 CTC cells. (**A**) High-throughput library screen: Experimental design overview. The hits inducing ≥ 50% decrease in viability are highlighted in red and indicated with an arrow. The dotted red line represents the threshold. (**B**) Effect of a 48 h treatment of BR16 cells with 250 compounds at a 5 μM concentration plotted as % of viability. Hit compounds leading to ≥50% reduction in viability are highlighted in red. (**C**) Schematic of the screening cascade.

With the aim of identifying compounds that reduce viability and result in growth inhibition of CTC-derived cells, we set up a drug-testing pipeline using a high-content imaging microscope. We employed our BR16 CTC-derived model, obtained from a patient with metastatic hormone-receptor positive breast cancer, and expanded it in suspension cultures and hypoxic (5% oxygen) conditions [10]. We used a dye combination to evaluate cellular viability and toxicity, consisting of Hoechst for nuclear staining and TOTO-3 for detecting dying cells. Viability was determined by subtracting the percentage of non-viable cells, identified as the proportion of TOTO-3-positive cells, in relation to the total count of Hoechst-positive nuclei within the same region. We screened 250 compounds in two sets of 82 and 168 compounds, respectively. The duplicate tests for each compound were conducted at a concentration of 5 μM, with an incubation time of 48 h (Figure 1A).

Our strategy successfully identified five compounds with marked activity against BR16 cells: US-464, US-463, DF-06, IIIM-368, and US-748 (Figure 1B,C). The identified hits were previously reported as CDK2/9 inhibitors [9], and their structure and CDK inhibitory activity is shown in Table 1.

**Figure 2:**
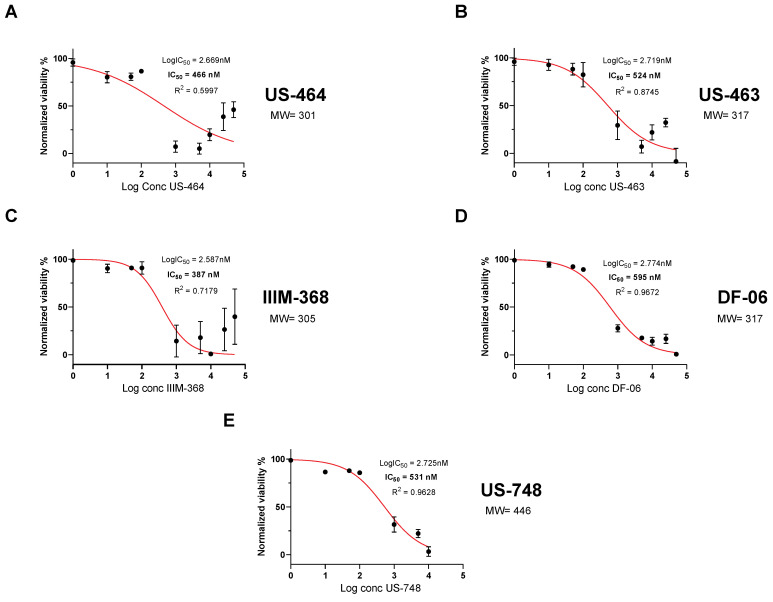
Hit compounds inhibit growth and reduce viability of BR-16 cells. Dose-dependent response (cell viability) following 48 h of treatment with US-464 (**A**), US-463 (**B**), IIIM-368 (**C**), DF-06 (**D**), and US-748 (**E**). Corresponding molecular weights for each indicated compound are shown.

To analyze dose-dependent effects on cell viability, we generated dose–response curves with our five hit compounds. Specifically, we determined an IC_50_ of 466 nM for US-464, 524 nM for US-463, 387 nM for IIM-368, 595 nM for DF-06, and 531 nM for US-748 (Figure 2).

These molecules were generated in multiple synthesis steps, starting from 5-bromoazaindole, and the details of chemical analysis and purification data of compounds are given in the Supplementary Data and in our earlier report [9]. We further validated the growth-inhibiting impact of chosen compounds in the murine 4T1 breast cancer cell line through an alternative MTT cytotoxicity assay (Supplementary Figure S1). We then investigated the impact of chosen compounds on the regulation of key cell cycle proteins in 4T1 cells. Given that in vitro effects of some of the identified compounds were already characterized in our previous study [9], we focused the present investigation on US-463, US-464, and DF-06. Specifically, we assessed their effects on the phosphorylation of cyclin-dependent kinase (CDK) targets using immunoblotting (Supplementary Figure S2). Our findings indicate that all three compounds significantly inhibited the phosphorylation of RNA polymerase II at the CDK9 site (p-Rpb1-CTD), which is crucial for transcriptional regulation of cell cycle regulation [18]. These compounds effectively suppressed the phosphorylation of retinoblastoma protein (p-Rb) at serine residues 807/811, a process regulated by CDK2 and CDK4. Inhibiting p-Rb phosphorylation disrupts cell cycle progression by blocking the G1/S phase transition. This preferentially dose-dependent disruption of key phosphorylation events suggests that these compounds may exert their anticancer effects through the inhibition of critical CDK-mediated pathways.

Together, our approach revealed cancer cytotoxic activity of five candidate molecules, to be further tested in mouse cancer models.

### 3.2. Transcriptome Profiling of BR16 Cells upon Treatment with Selected Compounds

**Figure 3:**
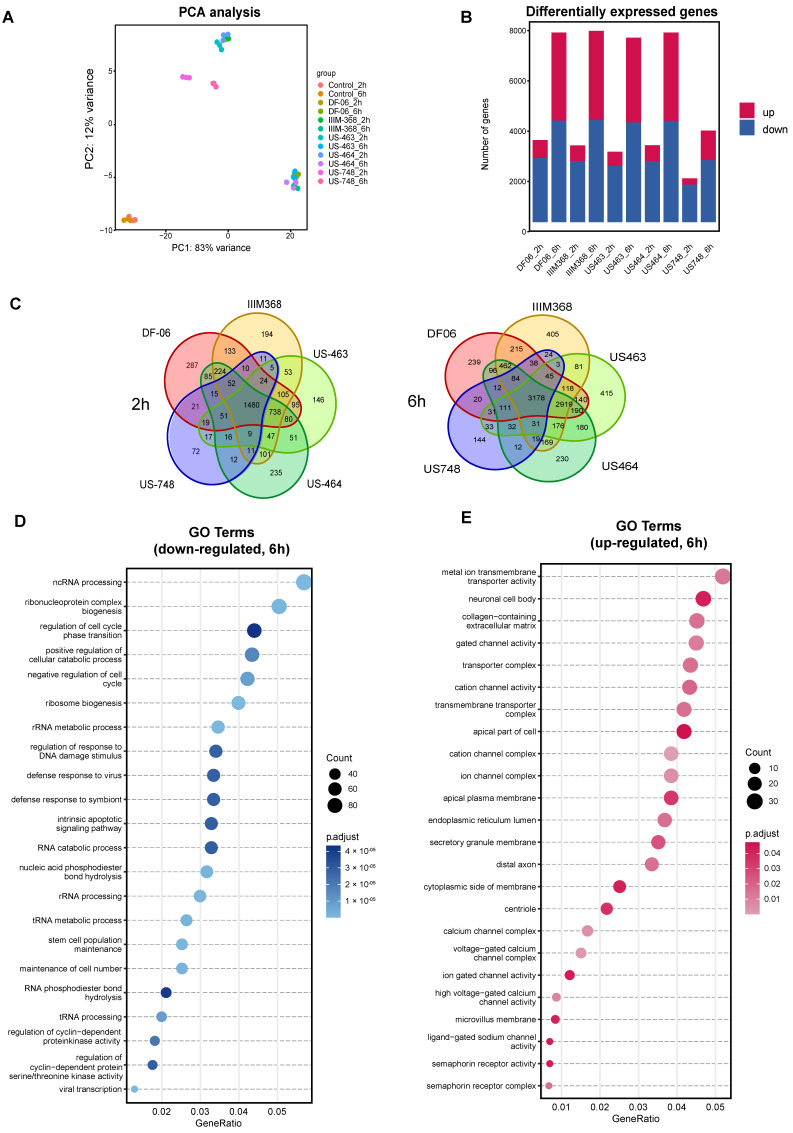
Analysis of BR16 cell transcriptome upon exposure to selected compounds. (**A**) Principal component analysis of BR16 cells treated with indicated compounds (5 µM) for two or six hours. (**B**) Quantification of differentially expressed genes (DEGs). (**C**) Venn diagram depicting the overlap of DEGs in indicated treatment groups. Gene Ontology enrichment analysis of common down-regulated (**D**) and up-regulated transcripts (**E**).

While some of the hit compounds were predicted kinase inhibitors based on our earlier studies [9], their antitumorigenic potential against breast CTC-derived cells and related mechanism of action were not previously explored. To further investigate the mechanistic aspects related to the activity of these compounds and to identify the pathways altered upon treatment, we performed RNA sequencing of BR16 CTC-derived cells treated with US-464, US-463, IIM-368, DF-06, and US-748 at 5 µM concentration for either two or six hours, in triplicate, and collected along with control cells. Principal component analysis revealed consistency among biological replicates and gene expression clusters of each group displayed distinct pre- and post-treatment transcriptional profiles (Figure 3A).

As expected, the number of differentially expressed genes (DEGs) compared to control cells increased along with the duration of treatment (Figure 3B, Supplementary Tables S1 and S2). The number of common DEGs in the five compound groups were 1480 and 3178 after two and six hours, respectively. To further dissect the cellular response to drug treatment, we carried out Gene Ontology (GO) enrichment analysis for the commonly down-regulated and up-regulated genes for all five compounds (Figure 3C,D). The analysis of down-regulated genes (Figure 3E) revealed an enrichment of genes related to the cell cycle, including those involved in the regulation of cell cycle phase transitions (e.g., *MYCN*, *RIPK1*, *SMAD1*), negative regulation of the cell cycle (e.g., *MDM2*, *SOX2*, *PLK2)*, and regulation of cyclin-dependent protein kinase activity (e.g., *PDGFB*, *CDKN2B*). Additionally, down-regulated genes exhibited enrichment for terms linked to stem cell population maintenance and RNA processing. In contrast, among the up-regulated genes, the most prevalent enriched terms were associated with ion channel complexes. Together, these results confirm cell cycle inhibition upon treatment with each of the five hit compounds, suggesting their suitability for further exploration of their in vivo activity.

### 3.3. In Vivo Effects of Hit Compounds in Mouse Models of Breast Cancer

**Figure 4:**
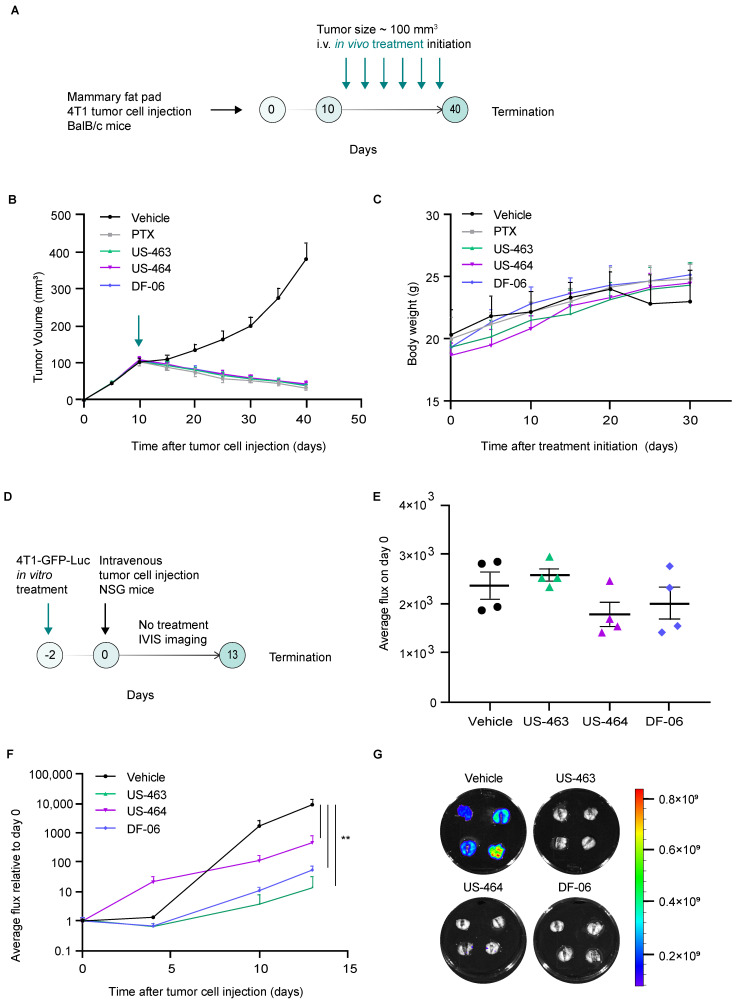
Hit compounds suppress primary tumor growth and metastasis in mouse models of breast cancer. (**A**) A schematic representation of the in vivo treatment study. (**B**) The impact of the specified compounds on the progression of primary tumor growth over time, with each group comprising six animals. The green arrow indicates the treatment initiation. (**C**) Monitoring of animal body weight following treatment initiation. (**D**) A schematic illustration of the experimental protocol involving in vitro treatment of the specified cells with the identified hit compounds, followed by an in vivo experimental metastasis study without treatment. (**E**) In vivo bioluminescence imaging of animals immediately upon tumor cell injection (day 0). (**F**) Real-time in vivo bioluminescence imaging of animals over the course of the study in the indicated treatment groups. (**G**) Images of ex vivo lung bioluminescent signals in specified experimental groups. **, *p* value < 0.01 by unpaired *t*-test.

Upon identification of promising cell cycle inhibitors in our assay, we then proceeded with testing their efficacy in preclinical mouse models of breast cancer. In vivo treatment studies investigating the effects of single agents (US-463, US-464, DF-06) on tumor development were conducted using the 4T1 orthotopic syngeneic mouse model. The in vivo efficacy of IIIM-368 has already been reported in our earlier studies [9]. Intravenous administration (twice a week) of individual compounds (15 mg/kg body weight), vehicle, or paclitaxel (used as positive control, 10 mg/kg body weight) for three weeks was initiated 10 days after 4T1 tumor cell injection into the mammary fat pad of Balb-c mice (Figure 4A).

Primary tumor growth was monitored by caliper measurements. All tested compounds displayed high therapeutic potential, reflected in significant inhibition of primary tumor growth in treated groups compared to the vehicle-treated control group (Figure 4B, Supplementary Figure S3). To investigate whether the reduced primary tumor size observed in treated mice could be attributed to decreased proliferative capacity, we examined the effects of the compounds on tumor cell proliferation using immunohistochemistry. Primary tumors sections were stained for Ki67, a widely used proliferation marker. Tumors from mice treated with the selected compounds exhibited markedly lower Ki67 expression compared with the vehicle-treated controls, demonstrating their anti-proliferative activity (Supplementary Figure S4). Of note, the overall antitumor effect was comparable between different compounds, which is in line with the fact that these small molecules belong to the same scaffold. The body weight of the compound-treated animals did not significantly change compared to the vehicle-treated mice (Figure 4C).

We subsequently evaluated whether compound treatment impacts metastasis by examining both migration in vitro and metastatic growth in vivo. Assessing migration in vitro allowed us to determine if the treatment alters early, motility-driven steps of dissemination, while the in vivo outgrowth model revealed whether these effects extend to later stages, such as seeding and expansion within a physiological environment. The wound healing assay demonstrated that treatment with selected compounds significantly impaired collective cell migration, indicating reduced migratory potential (Supplementary Figure S5). Next, we explored whether compound treatment—using concentrations corresponding to the IC_50_ for a duration of 48 h—would lead to a reduced proficiency of the treated cells to seed metastatic lesions in vivo upon injection in untreated mice. We employed two complementary experimental approaches: a short-term study to assess early metastatic seeding and proliferative capacity, and a long-term study to evaluate metastatic outgrowth over time. In the short-term approach, 4T1 cells stably expressing GFP and Luc were pretreated with the selected compounds and injected into the tail vein of NSG mice, and their lungs were harvested 24 h post-injection (Supplementary Figure S6). Ki67 expression was assessed by immunofluorescence staining of lung sections, revealing a significant reduction in the percentage of Ki67-positive tumor cells in mice injected with compound-pretreated cells compared to the controls, indicating impaired proliferative activity. In the long-term approach, upon treatment with selected compounds, 4T1 cells stably expressing GFP and Luc were injected into the tail vein of NSG mice, and a non-invasive in vivo monitoring schedule through bioluminescence imaging was used to investigate their ability to seed and propagate metastatic lesions over time (Figure 4D). Our findings reveal that the treatment with US-464, US-463, or DF-06 had no immediate impact on the ability of 4T1-GFP-Luc cells to establish themselves in lung tissue directly after injection at day 0 (Figure 4E). However, upon experiment termination, the bioluminescent signal was significantly lower in all treatment groups compared to the control group of animals (Figure 4F,G). These results demonstrate that the ability of 4T1 cells to expand within the lungs—a major metastatic site—is impaired by in vitro drug exposure even in the absence of subsequent in vivo treatment.

## 4. Discussion

Even though more than 90% of cancer associated deaths are attributed to metastasis, currently there are no available therapies to circumvent a metastatic disease. Recently, CTC enumeration in patient blood has been shown to correlate with therapeutic efficacy [19]. Cancer-specific CTC lines with metastatic abilities offer an excellent in vitro predictive and drug screen model to discover new therapeutic agents. Previous investigations have used patient-derived CTCs of various cancers as drug testing models for anticancer agents [7,19,20], demonstrating their potential as a tool for personalized medicine.

Our current study employed CTC-based BR16 line to screen a 250 small-molecule library that led to the identification of five hits, all belonging to the marine scaffolds bearing strong CDK2 and CDK9 inhibitory activities. While this study focuses on CDK2/9 inhibition, potential effects on CDK4 were not examined and remain an area for future investigation. Earlier, marine-derived CDK4 inhibitor fascaplysin was tested against a panel of lung cancer-derived CTC lines, showing its varied sensitivity among these lines [21]. Given the well-known role of CDKs in cancer progression, extensive efforts have been made to develop efficient and specific CDK inhibitors as valuable therapeutic options. Over the last three decades, numerous agents targeting CDKs have been tested across different phases of clinical trials [22]. The combinatorial use of CDK inhibitors with chemotherapy have shown promising clinical results with manageable toxicity and side effects in breast cancer. Particularly, the discovery and development of CDK4/6 inhibitors has provided a significant addition to the field, including molecules like palbociclib, ribociclib, abemaciclib, and trilaciclib, which have been approved by the FDA for the treatment of various cancer types [23]. Oral CDK4/6 inhibitors (palbociclib, ribociclib, and abemaciclib) are approved as first-line therapy used in combination with either an aromatase inhibitor or fulvestrant for hormone receptor-positive and human epidermal growth factor receptor 2 (HER2)-negative metastatic breast cancer (extensively reviewed in [24]). Along these lines, palbociclib, a CDK4/6 inhibitor, exhibited promising activity against breast cancer-derived CTC line CTC-ITB-01, which mimicked clinical features of metastasis in xenograft models [25].

Our small-molecule screen, for the first time, identified and validated new CDK2/9 inhibitors effective against CTC-derived breast cancer cells. Recently, targeting CDK2 and CDK9 has emerged as a potential approach for cancer therapy [26,27]. The advancement of first-generation CDK9 inhibitors like alvocidib and dinaciclib has been impeded by toxicity and adverse effects, most likely stemming from their pan-CDK inhibition activity [28]. Second-generation inhibitors, such as BAY1251152 and AZD4573, are presently undergoing clinical trials in patients with solid tumors and hematological malignancies, offering improved safety profiles [28,29]. The FDA has granted fast-track designation and orphan drug designation to SLS009, a new CDK9 inhibitor, for the treatment of patients with relapsed or refractory peripheral T-cell lymphoma and acute myeloid leukemia, respectively, based on the NCT04588922 (https://clinicaltrials.gov/study/NCT04588922) clinical trial. Another oral CDK9 inhibitor, KB-0742, is being evaluated in phase I/II studies in relapsed solid tumors, including breast cancer (NCT04718675 (https://clinicaltrials.gov/study/NCT04718675?viewType=Table&cond=CDK9&rank=2)). CDK2 inhibitors currently tested in phase I studies in breast cancer patients include INX-315 (NCT05735080 (https://clinicaltrials.gov/study/NCT05735080?viewType=Table&cond=CDK2&page=1&rank=3)), BG-68501 (NCT06257264 (https://clinicaltrials.gov/study/NCT06257264?viewType=Table&cond=CDK2&page=1&rank=4)), BLU-22 (NCT05252416 (https://clinicaltrials.gov/study/NCT05252416?viewType=Table&cond=CDK2&page=1&rank=7)), NKT3447 (NCT06264921 (https://clinicaltrials.gov/study/NCT06264921?viewType=Table&cond=CDK2&page=1&rank=8)), and PF-07104091 (NCT05262400 (https://clinicaltrials.gov/study/NCT05262400?viewType=Table&cond=CDK2&page=2&rank=11)).

By employing a combination of our drug screen platform, in vivo models of metastatic breast cancer, RNA sequencing, and in vitro cell assays, our study resulted in the identification of CDK2/9 inhibitors that are active in the reduction of tumor growth and lung colonization in breast cancer, with no observed adverse effects or toxicity. Furthermore, our transcriptomic data delineate that metastasis-relevant signaling pathways are affected by these treatments, including pathways involved in cyclin-dependent protein kinase activity.

## 5. Conclusions

In conclusion, our study identified novel small-molecule CDK2/9 inhibitors with a potent anti-cancer activity in breast cancer, warranting further preclinical development.

## Figures and Tables

**Table 1:** Structure of identified hits and their CDK2/9 inhibitory activity.

Name	Structure	Kinase Activity	
		CDK2 (IC_50_, nM)	CDK9 (IC_50_, nM)
IIIM-368	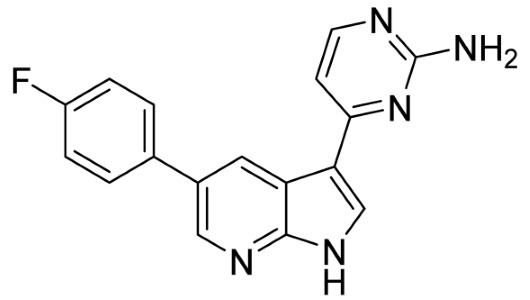	5.5	24
US-463	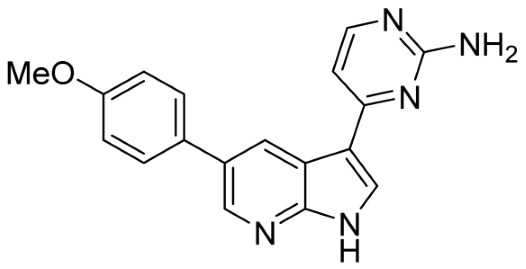	6	28
US-464	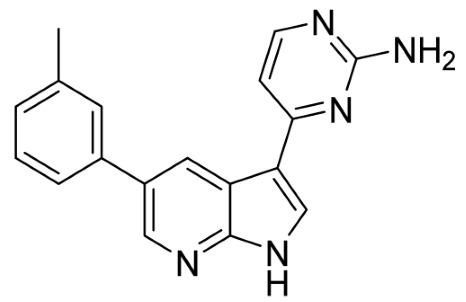	92	146
DF-06	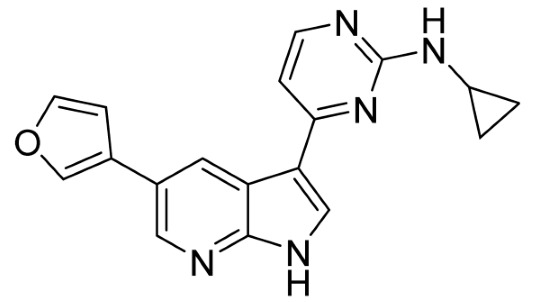	84	90
US-748	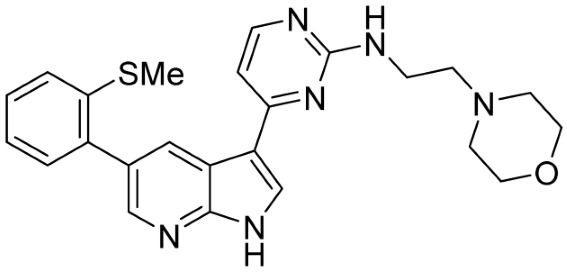	117	172

## Data Availability

RNA sequencing data have been deposited to Gene Expression Omnibus [30], under accession number GSE251771.

## Supplementary Materials

The following supporting information can be downloaded at https://www.mdpi.com/article/10.3390/cancers18030466/s1: Supplementary Data: Details of chemical analysis and purification data of compounds and original images of uncropped Western blots. Supplementary Scheme S1: Synthetic strategy for the generation of DF-06 and US-748. Supplementary Figure S1: Dose-dependent response (MTT assay) following 48 h treatment with US-463, US-464, or DF-06 in 4T1-GFP-luciferase breast cancer cell line. Supplementary Figure S2: Western blot analysis of whole-cell extracts from 4T1 cells left untreated or treated for 12 h with the indicated concentrations of the specified compounds and probed with the indicated antibodies. Corresponding quantification is shown. Supplementary Figure S3: Effects of hit compounds on final primary tumor weight. (A) Photographs of harvested primary tumors from three out of six animals treated with indicated compounds. (B) A dot blot diagram depicting final primary tumor weight in specified animal groups. Supplementary Figure S4: Representative immunohistochemistry images (10× and 40×) showing Ki67 protein expression in 4T1 primary tumors isolated from animals treated with vehicle or the indicated compounds. Scale bars: 100 µm (10×) and 50 µm (40×). Supplementary Figure S5: Effects of the chosen compounds on 4T1 cell migration assessed by wound healing assay. Representative images (top) of wounds in 4T1 cell monolayers captured immediately after scratching and after 16 h of incubation with control (0.25% DMSO), US-463 (102 nM), US-464 (138 nM), or DF-06 (792 nM), along with corresponding quantification (bottom). *, *p* value < 0.05; **, *p* value < 0.01 by unpaired *t*-test. Scale bar, 20 µm. Supplementary Figure S6: Representative immunofluorescence images showing Ki67 protein expression in 4T1 lung samples isolated from animals intravenously injected with cell in vitro pre-treated with vehicle or the indicated compounds (left) and quantification (right). Scale bar, 20 µm. Supplementary Table S1: List of all down-regulated genes in BR16 cells upon treatment with cells treated with indicated compounds (5 µM) for two or six hours. Supplementary Table S2: List of all up-regulated genes in BR16 cells upon treatment with cells treated with indicated compounds (5 µM) for two or six hours.
