# Supplementary material for: Novel Cell Cycle Inhibitors Decrease Primary and Metastatic Breast Cancer Growth In Vivo
Source: Cancers (Basel). 2026 Jan 30;18(3):466. doi: 10.3390/cancers18030466 (PMC12896542; doi:10.3390/cancers18030466)
Supplement: Supplementary file 1 [file cancers-18-00466-s001.zip › supplementary figures.pdf]

A

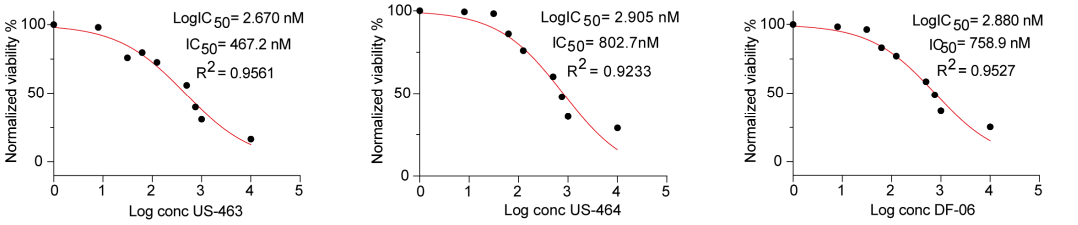

B

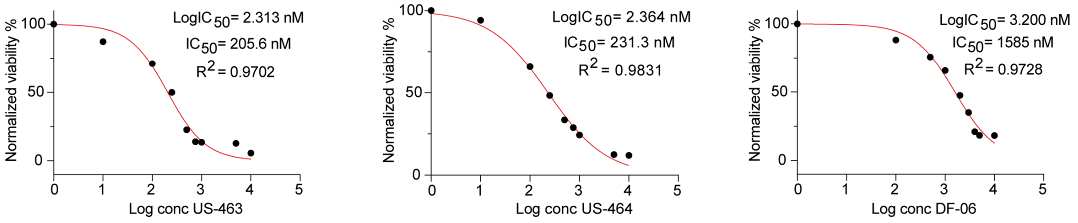

Supplementary Figure S1: Dose-dependent response (MTT assay) following 48 h treatment with US-463, US-464, or DF-06 in 4T1-GFP-luciferase breast cancer cell line.

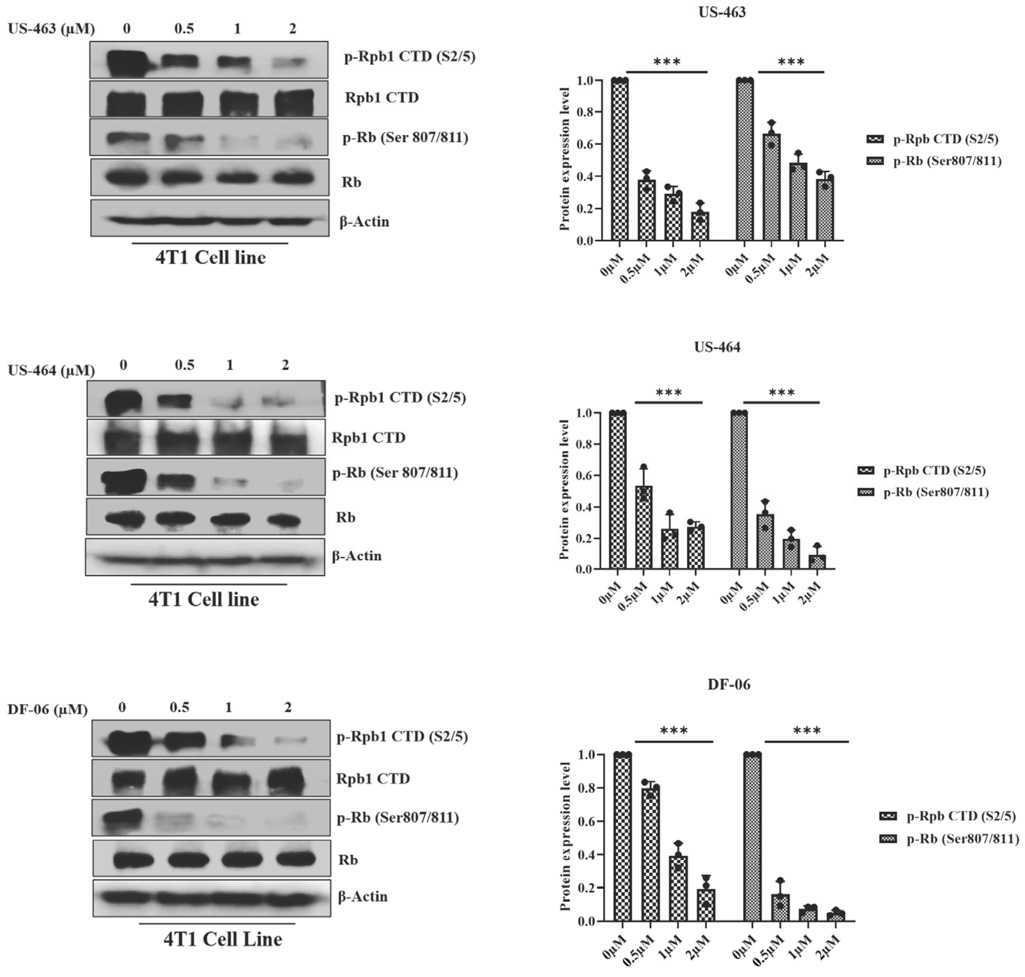

Supplementary Figure S2: Western blot analysis of whole-cell extracts from 4T1 cells left untreated or treated for 12 h with the indicated concentrations of the specified compounds and probed with the indicated antibodies. Corresponding quantification is shown.

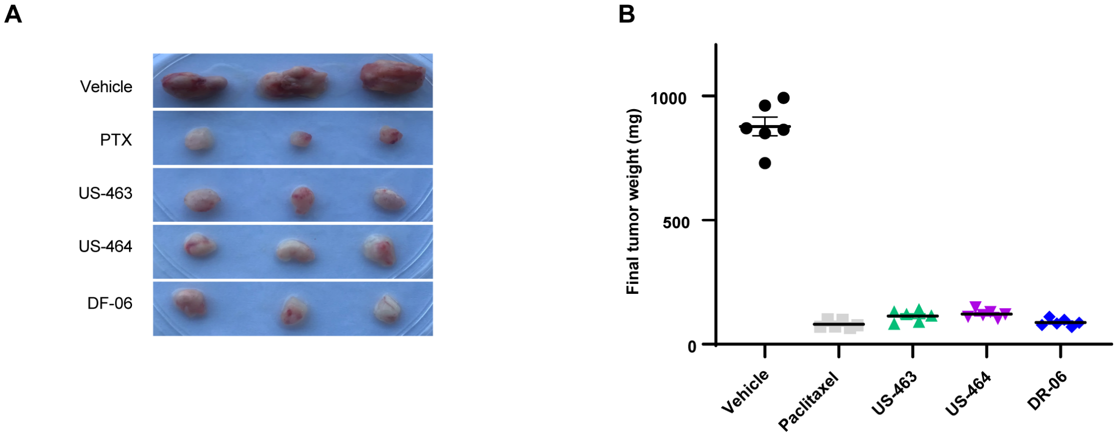

Supplementary Figure S3: Effects of hit compounds on final primary tumor weight. (A) Photographs of harvested primary tumors from three out of six animals treated with indicated compounds. (B) A dot blot diagram depicting final primary tumor weight in specified animal groups.

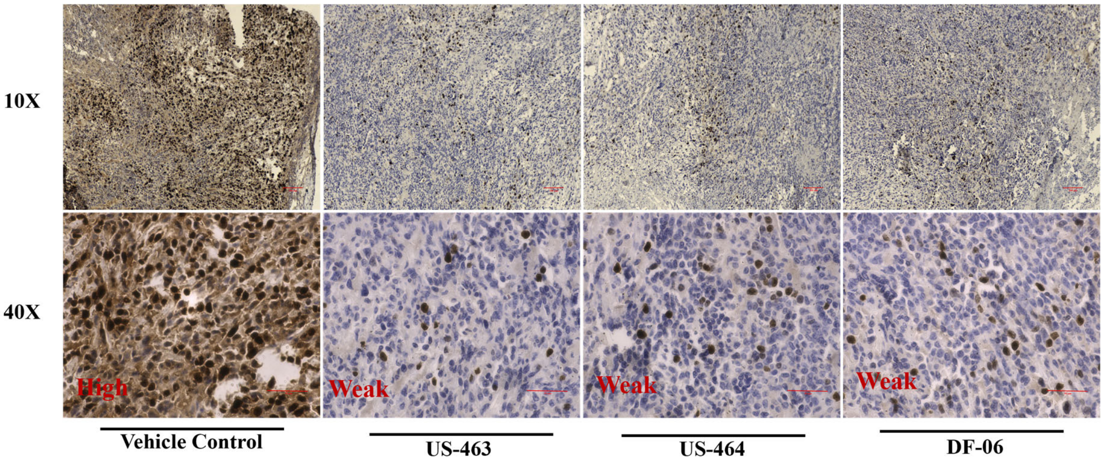

Supplementary Figure S4: Representative immunohistochemistry images (10× and 40×) showing Ki67 protein expression in 4T1 primary tumors isolated from animals treated with vehicle or the indicated compounds. Scale bars: 100 µm (10×) and 50 µm (40×).

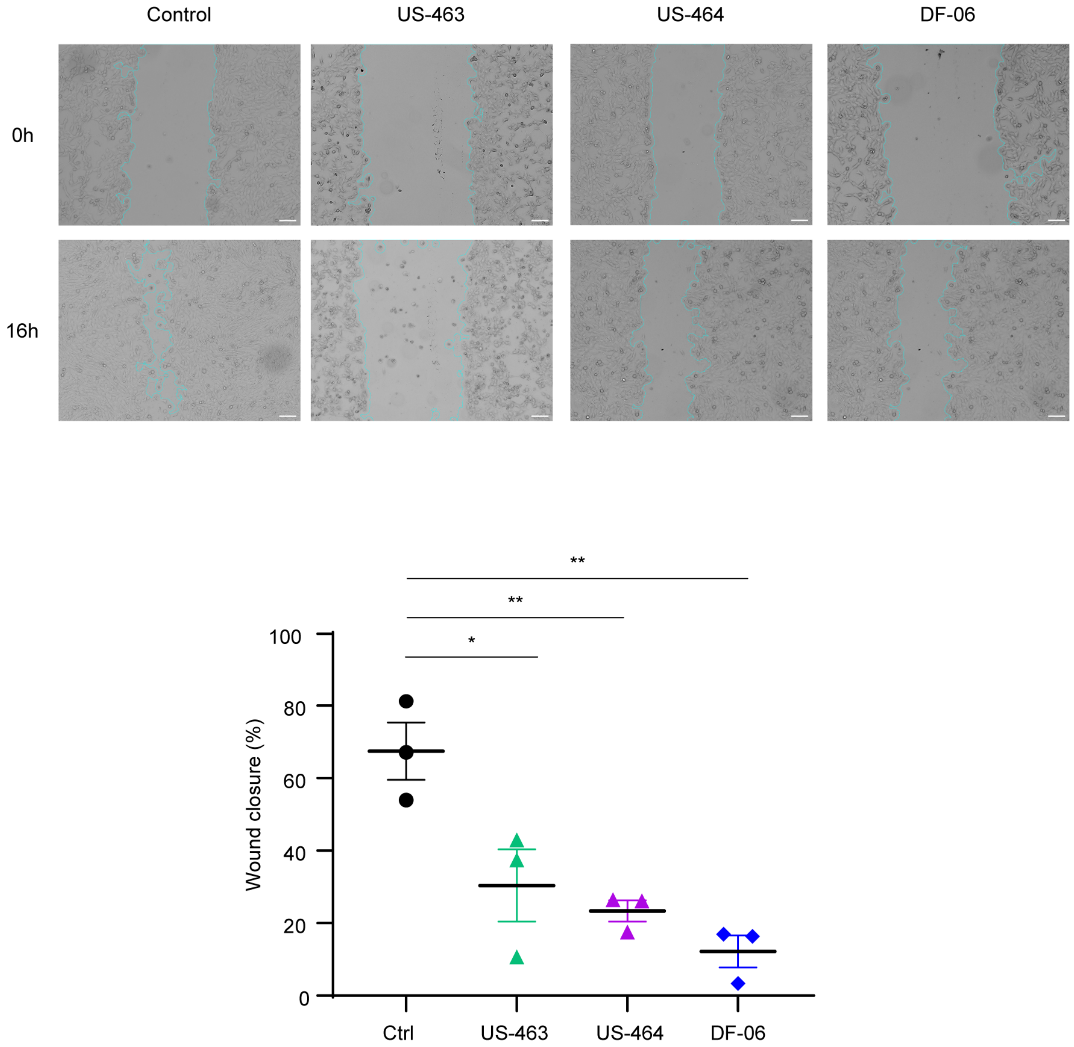

Supplementary Figure S5: Effects of the chosen compounds on 4T1 cell migration assessed by wound healing assay. Representative images (top) of wounds in 4T1 cell monolayers captured immediately after scratching and after 16 h of incubation with control (0.25% DMSO), US-463 (102 nM), US-464 (138 nM), or DF-06 (792 nM), along with corresponding quantification (bottom). *, p value < 0.05; **, p value < 0.01 by unpaired t-test. Scale bar, 20 μm.

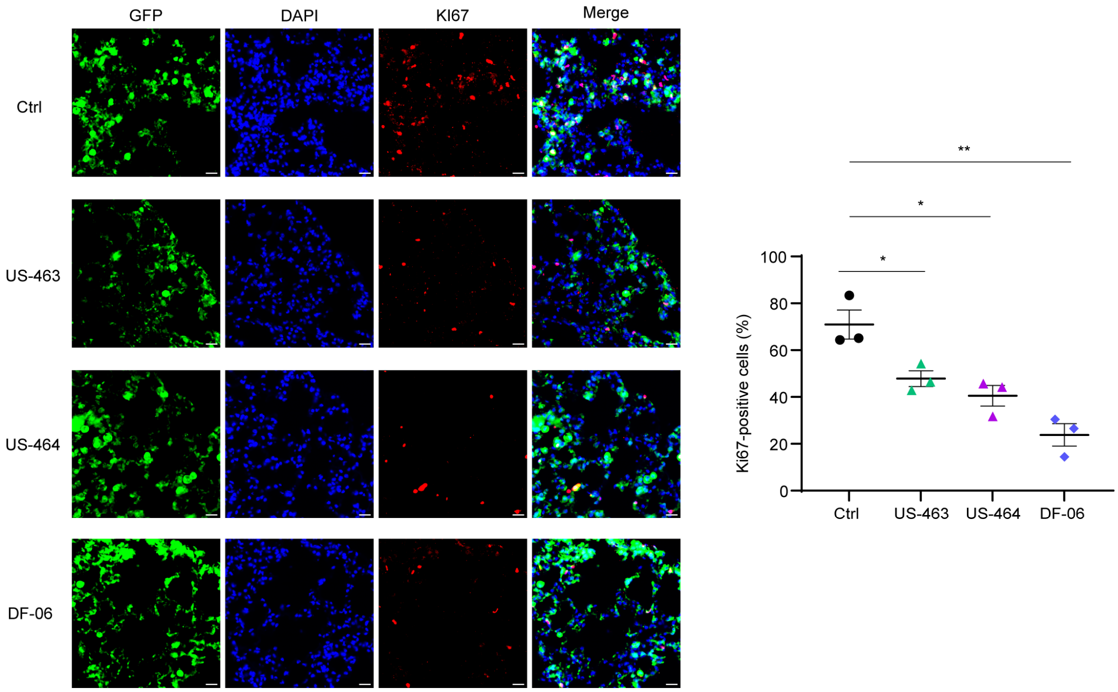

Supplementary Figure S6: Representative immunofluorescence images showing Ki67 protein expression in 4T1 lung samples isolated from animals intravenously injected with cell in vitro pre-treated with vehicle or the indicated compounds (left) and quantification (right). Scale bar, 20 μm.
