# Supplementary material for: Novel Cell Cycle Inhibitors Decrease Primary and Metastatic Breast Cancer Growth In Vivo
Source: Cancers (Basel). 2026 Jan 30;18(3):466. doi: 10.3390/cancers18030466 (PMC12896542; doi:10.3390/cancers18030466)
Supplement: Supplementary file 1 [file cancers-18-00466-s001.zip › Supplementary Data.pdf]

Supplementary Data

Table of Contents

1.	General Scheme	3
2.	Synthesis of intermediate 3	3-4
3.	Synthesis of intermediate 4-Chloro-N-(2-morpholinoethyl)pyrimidin-2-amine (4)	4-5
4.	Synthesis of final compound DF-06	5-6
5.	Synthesis of final compound US-748	6-7
6.	1H NMR, 13C-NMR, HRMS spectras of compounds DF-06	8-9
7.	1H NMR, 13C-NMR, HRMS spectras of compounds US-748	10-11
8.	Original images of Western Blots	12-14

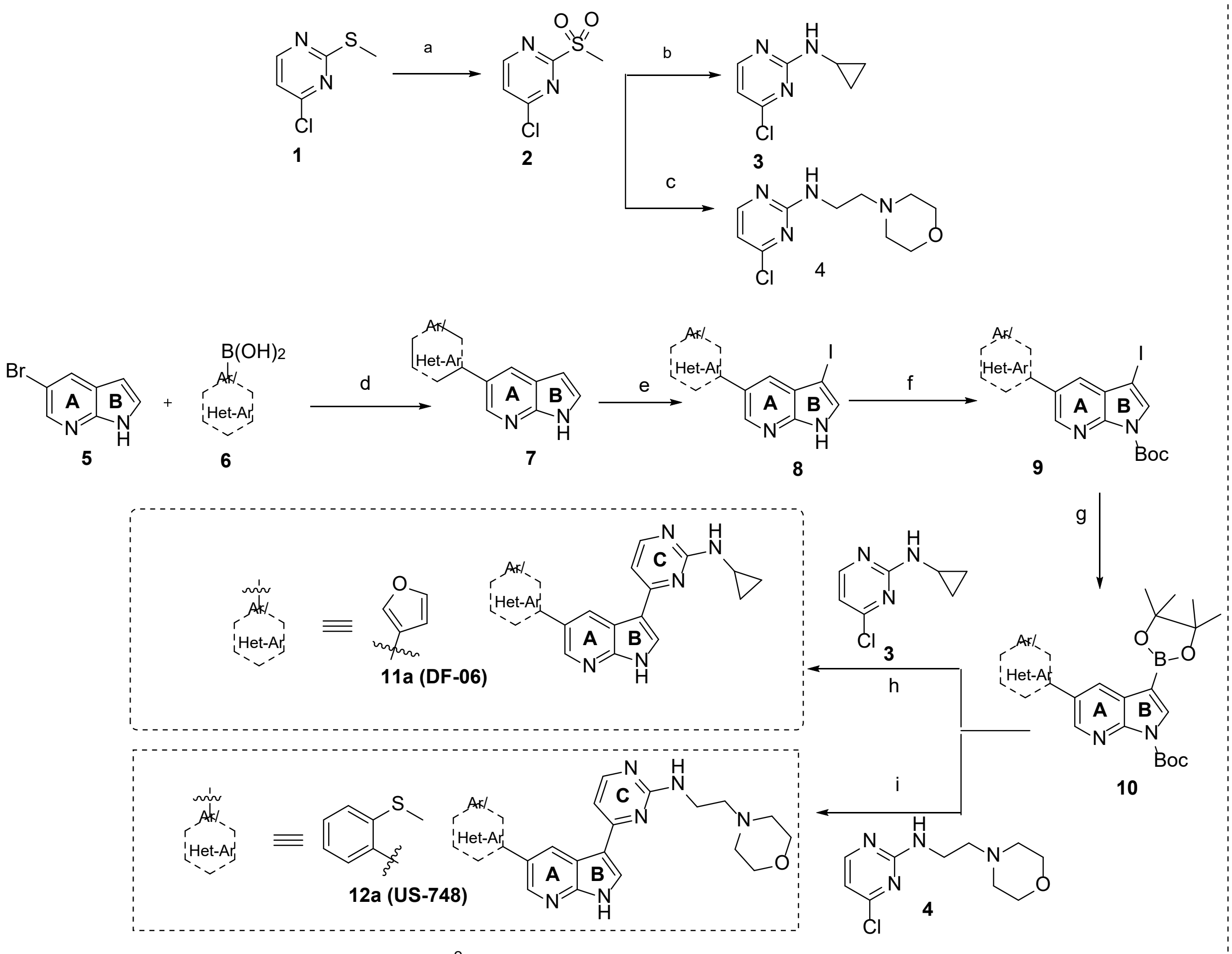

Reagents and conditions : (*a*) *m*-CPBA, CH_2Cl_2, 0 ^oC - rt, 2 h; (b) Cyclopropyl amine, THF, rt, 2 h; (c) ethylmorpholylamine, THF, rt, 2 h; (d) $PdCl_2$(dppf), K_2CO_3, dioxane:H_2O, 80 ^oC, 6-12 h; (e) I_2, KOH, DMF, rt, 0.5-1 h; (f) Boc anhydride, DMAP, DCM, rt, 0.5-1 h; (g). $Pd(PPh_3)_4$, anhydrous Et_3N, anhydrous dioxane, HBPin, 80 ^oC, 3-4 h; (h) $Pd(PPh_3)_4$, **3**, Cs_2CO_3, anhydrous MeOH, 100 ^oC, 35-49 h; (i) $Pd(PPh_3)_4$, **4**, Cs_2CO_3, anhydrous MeOH, 100 ^oC, 35-49 h.

Scheme S1: Synthetic strategy for the generation of DF-06 and US-748

Synthesis of intermediates:

i) **Synthesis of 4-Chloro-*N*-cyclopropylpyrimidin-2-amine (3):** *meta*-Chloroperbenzoic acid (*m*-CPBA) (1.5 mmol) was added portion wise to an ice cooled solution of **1** (1.0

mmol) in dichloromethane and resulting reaction mixture was stirred at 25 °C for 2 h. After completion of reaction, monitored by thin layer chromatography and reaction mixture was washed with saturated solution of sodium bicarbonate, extracted with dichloromethane and concentrated in- vaccuo to obtain beige solid **2**, which was used without further purification for the next step. To the solution of **2** in tetrahydrofuran, cyclopropyl amine (2.0 equiv) was added and resulting reaction mixture was stirred at room temperature for 2 h and solvents were removed in vaccuo and the residue was absorbed onto celite and purified chromatographically on silica gel with ethyl acetate/hexane system to obtained pure compounds **3**.TLC (EtOAC:Hexane 2:8) R_f = 0.6; Color/nature: white/solid; ^{1}H NMR (400 MHz, $CDCl_3$) δ 8.20 (d, J = 5.0 Hz, 1H), 6.61 (d, J = 5.2 Hz, 1H), 5.56 (s, 1H), 2.82 – 2.76 (m, 1H), 0.92 – 0.73 (m, 2H), 0.62 – 0.45 (m, 2H); ^{13}C NMR (101 MHz, $CDCl_3$) δ 162.8, 160.7, 158.5, 110.0, 23.4, 6.8; DEPT NMR (101 MHz, $CDCl_3$) δ 159.0, 110.5, 23.9, 7.3; HRMS (ESI-TOF) calc'd for $C_7H_9ClN_3$ [M + H^+] 170.0485; found 170.0490.

ii) Synthesis of intermediate 4-Chloro-*N*-(2-morpholinoethyl)pyrimidin-2-amine (4): *meta*-Chloroperbenzoic acid (*m*-CPBA) (1.5 mmol) was added portion wise to an ice cooled solution of **1** (1.0 mmol) in dichloromethane and resulting reaction mixture was stirred at 25 °C for 2 h. After completion of reaction, monitored by thin layer chromatography and reaction mixture was washed with saturated solution of sodium bicarbonate, extracted with dichloromethane and concentrated in- vaccuo to obtain beige solid **2**, which was used without further purification for the next step. To the solution of **2** in tetrahydrofuran, ethylmorpholylamine (2.0 equiv) was added and resulting reaction mixture was stirred at room temperature for 2 h and solvents were removed in vaccuo and the residue was absorbed onto celite and purified chromatographically on silica gel with ethyl acetate/hexane system to obtained pure compounds (**4**). TLC (MeOH: DCM 5:95) R_f = 0.6; Color/nature: oily liquid; ^{1}H NMR (400 MHz, $CDCl_3$) δ 8.14 (d, J = 4.1 Hz, 1H), 6.55 (d, J = 5.2 Hz, 1H), 6.12 (s, 1H), 3.76 – 3.61 (m, 4H), 3.51 (dd, J = 11.4, 5.7 Hz, 2H), 2.58 (t, J = 6.0 Hz, 2H), 2.49 (s, 4H); ^{13}C NMR (126 MHz, $CDCl_3$) δ 162.2, 161.2, 159.0, 109.8, 66.8, 56.9, 53.3, 37.6; DEPT NMR (126 MHz, $CDCl_3$) δ 158.5, 109.3, 66.3, 56.4, 52.8, 37.1; HRMS (ESI-TOF) calc'd for $C_{10}H_{16}ClN_4O$ [M + H^+] 243.1013; found 243.1013.

iii) Synthesis of final compound 11a (DF-06): Tetrakis (triphenylphosphane)-palladium (0) (3 mol %) and key intermediates 5-substituted *tert*-butyl 3-iodo-1*H*-pyrrolo[2,3-*b*]pyridine-1-carboxylate **9** (1.00 mmol) were placed under argon atmosphere in a dry screw-cap vessel with septum. Then, 5 mL of dry dioxane were added and the mixture was degassed with argon. Dry triethylamine (10.0 mmol, 10.0 equiv), and 4,4,5,5-tetramethyl-1,3,2-dioxaborolane (1.50 mmol, 1.50 equiv) were successively added to the mixture which was stirred at 80 °C (preheated oil bath) for 3-4 h to obtain **10** (monitored by TLC). Then, after cooling to at 25 ºC, Tetrakis (triphenylphosphane)-palladium (0) (3 mol %), 5 mL of dry methanol, 1.00 mmol of 4-chloro-*N*-cyclopropylpyrimidin-2-amine (**3**) and cesium carbonate (2.50 mmol, 2.50 equiv) were successively added and the mixture was stirred at 100 °C for 35-49 h. Then, after cooling at 25 ºC the solvents were removed in vaccuo and the residue was absorbed onto Celite and purified chromatographically on silica gel with dichloromethane-methanol-aqueous ammonia (isocratic or stepwise gradient). The obtained bis(hetero)aryls **11a** can be further purified by suspending in dichloromethane, sonication in ultrasound bath for 0.5-1.0 h, filtration and drying in vaccuo overnight for 12 h to obtained the compounds of formula **11a** or **DF-06** (**Note**: Procedure for synthesis of all other intermediates and final compounds (IIIM-368, US-463 and US-464) reported in *J. Med. Chem.* **2017**, *60*, 9470-9489). TLC (MeOH: DCM 1:9) R_f = 0.4; Yield: 35 %; Color/nature: yellow/solid; m.p. = 277-279 ºC; ^{1}H NMR (400 MHz, DMSO-d_6) δ 12.25 (s, 1H), 9.09 (s, 1H), 8.59 (d, J = 2.1 Hz, 1H), 8.39 (d, J = 2.7 Hz, 1H), 8.21 (d, J = 5.3 Hz, 2H), 7.80 (t, J = 1.7 Hz, 1H), 7.30 (s, 1H), 7.14 (d, J = 5.3 Hz, 1H), 1.28 – 1.18 (m, 1H), 0.78 – 0.71 (m, 2H), 0.60 – 0.52 (m, 2H); ^{13}C NMR (126 MHz, DMSO-d_6) δ 161.9, 160.5, 155.6, 147.1, 143.0, 140.3, 137.3, 127.9, 125.9, 122.9, 120.0, 116.5, 111.2, 107.6, 103.8, 22.5, 5.3; HRMS (ESI-TOF) calc'd for $C_{18}H_{16}N_5O$ [M + H$^+$] 318.1355; found 318.1355; HPLC-purity 98 % (t_R = 32.02).

iv) Synthesis of final compounds 12a (US-748): Tetrakis (triphenylphosphane)-palladium (0) (3 mol %) and key intermediates 5-substituted *tert*-butyl 3-iodo-1*H*-pyrrolo[2,3-*b*]pyridine-1-carboxylate (**9**) (1.00 mmol) were placed under argon atmosphere in a dry screw-cap vessel with septum. Then, 5 mL of dry dioxane were added and the mixture was degassed with argon. Dry triethylamine (10.0 mmol, 10.0 equiv), and 4,4,5,5-tetramethyl-1,3,2-dioxaborolane (1.50 mmol, 1.50 equiv) were successively added to the mixture which was

stirred at 80 °C (preheated oil bath) for 3-4 h to obtain **10** (monitored by TLC). Then, after cooling to at 25 ºC, Tetrakis (triphenylphosphane)-palladium (0) (3 mol %), 5 mL of dry methanol, 1.00 mmol of 4-Chloro-*N*-(2-morpholinoethyl)pyrimidin-2-amine (**4**) and cesium carbonate (2.50 mmol, 2.50 equiv) were successively added and the mixture was stirred at 100 °C for 35-49 h. Then, after cooling at 25 ^{0}C (water bath) the solvents were removed in vaccuo and the residue was absorbed onto Celite and purified chromatographically on silica gel with dichloromethane-methanol-aqueous ammonia (isocratic or stepwise gradient). The obtained bis(hetero)aryls **12a** can be further purified by suspending in dichloromethane, sonication in ultrasound bath for 0.5-1.0 h, filtration and drying in vaccuo overnight for 12 h to obtain the compound **12a (US-748)**. TLC (MeOH: DCM 1:9) R_f = 0.5; Yield: 44 %; Color/nature: yellow/solid; m.p. = 236-238 ºC; ^{1}H NMR (400 MHz, DMSO) δ 12.35 (s, 1H), 8.90 (s, 1H), 8.44 (d, *J* = 1.7 Hz, 1H), 8.23 (d, *J* = 1.7 Hz, 1H), 8.16 (d, *J* = 5.2 Hz, 1H), 7.47 – 7.34 (m, 2H), 7.34 – 7.23 (m, 2H), 7.08 (d, *J* = 5.3 Hz, 1H), 6.87 (t, *J* = 5.1 Hz, 1H), 3.51– 2.45 (m, 12H, merge with peak of DMSO-d_6), 2.46 (s, 3H); ^{13}C NMR (126 MHz, DMSO-d_6) δ 162.7, 149.2, 142.8, 137.5, 135.9, 133.7, 130.2, 129.6, 128.8, 128.0 (2C), 127.0 (3C), 118.3, 113.6, 105.2, 66.2, 57.2, 52.8, 15.2; HRMS (ESI-TOF) calc'd for $C_{24}H_{27}N_6OS$ [M + H^{+}] 447.1967;found 447.1967; HPLC-purity 97 % (t_R = 26.3).

^{1}H-NMR of DF-06:

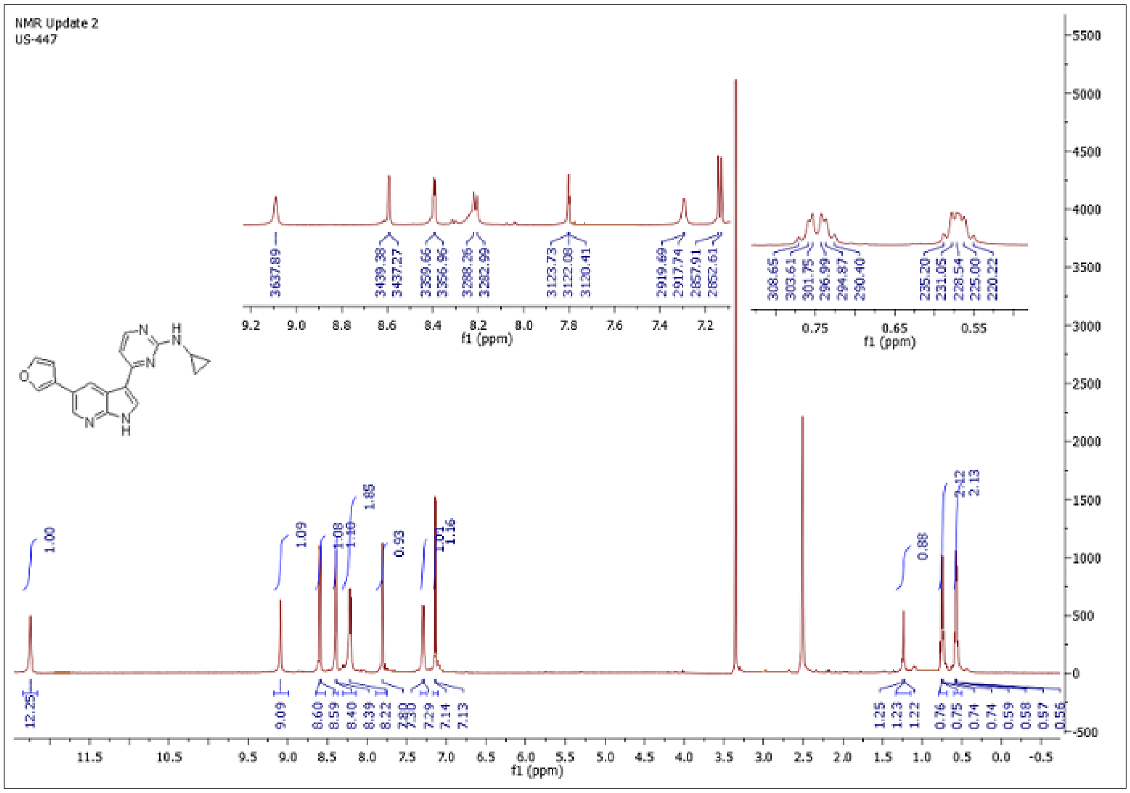

^{13}C-NMR of DF-06:

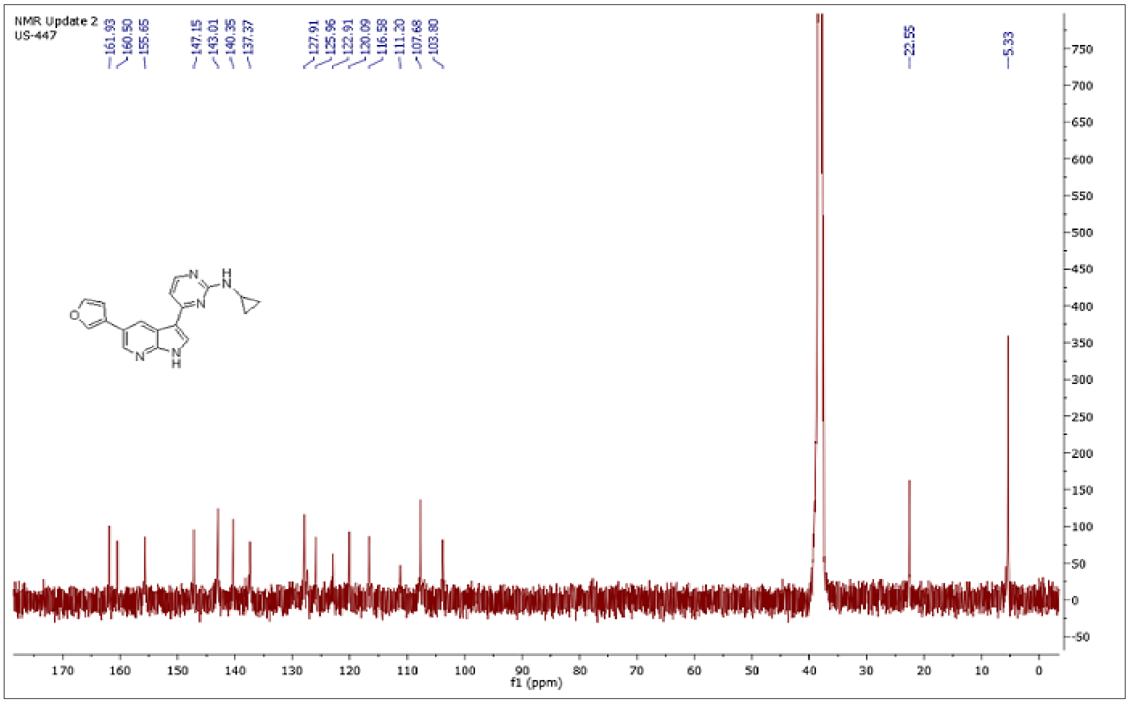
NMR Update 2
US-447
161.93
160.50
155.65
147.15
143.01
140.35
137.37
127.91
125.96
122.91
120.09
116.58
111.20
107.68
103.80
22.55
5.33
f1 (ppm)

HRMS of DF-06:

Qualitative Compound Report

**Data File**	US-447.d	**Sample Name**	US-447
**Sample Type**	Sample	**Position**	Vial 12
**Instrument Name**	Instrument 1	**User Name**	
**Acq Method**	new method.m	**Acquired Time**	16-05-2014 PM 1:40:24
**IRM Calibration Status**	Success	**DA Method**	daily_report.m
**Comment**			

Sample Group **Info.**
Acquisition SW Version 6200 series TOF/6500 series Q-TOF B.05.01 (B5125)

Compound Table

Compound Label	RT	Mass	Formula	MFG Formula	MFG Diff (ppm)	DB Formula
Cpd 41: C18 H15 N5 O	0.264	317.1283	C18 H15 N5 O	C18 H15 N5 O	-2.06	C18 H15 N5 O

Compound Label	*m/z*	RT	Algorithm	Mass
Cpd 41: C18 H15 N5 O	318.1355	0.264	Find by Molecular Feature	317.1283

MFE MS Spectrum

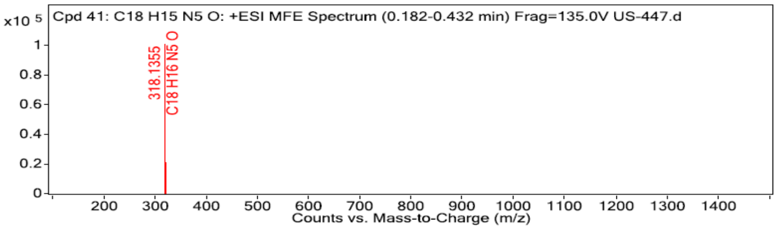

MS Spectrum Peak List

m/z	z	Abund	Formula	Ion
318.1355	1	100919.86	C18 H16 N5 O	(M+H)+
319.1388	1	21527.91	C18 H16 N5 O	(M+H)+
320.1413	1	2688.99	C18 H16 N5 O	(M+H)+
321.1417	1	588.08	C18 H16 N5 O	(M+H)+

Predicted Isotope Match Table

Isotope	*m/z*	Calc m/z	Diff (ppm)	Abund %	Calc Abund %	Abund Sum %	Calc Abund Sum %
1	318.1355	318.1349	-1.84	100	100	80.27	80.57
2	319.1388	319.1378	-3.16	21.33	21.52	17.12	17.34
3	320.1413	320.1405	-2.63	2.66	2.41	2.14	1.94
4	321.1417	321.1431	4.38	0.58	0.19	0.47	0.15

--- End Of Report ---

^{1}H-NMR of US-748

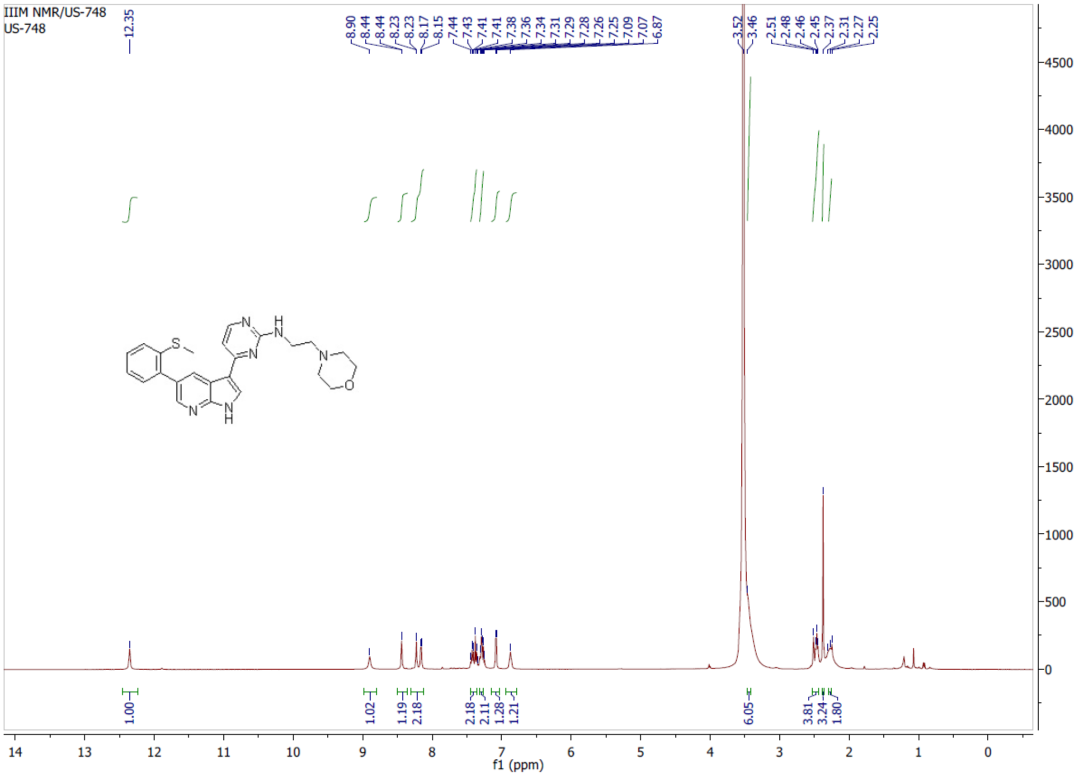

^{13}C-NMR of US-748:

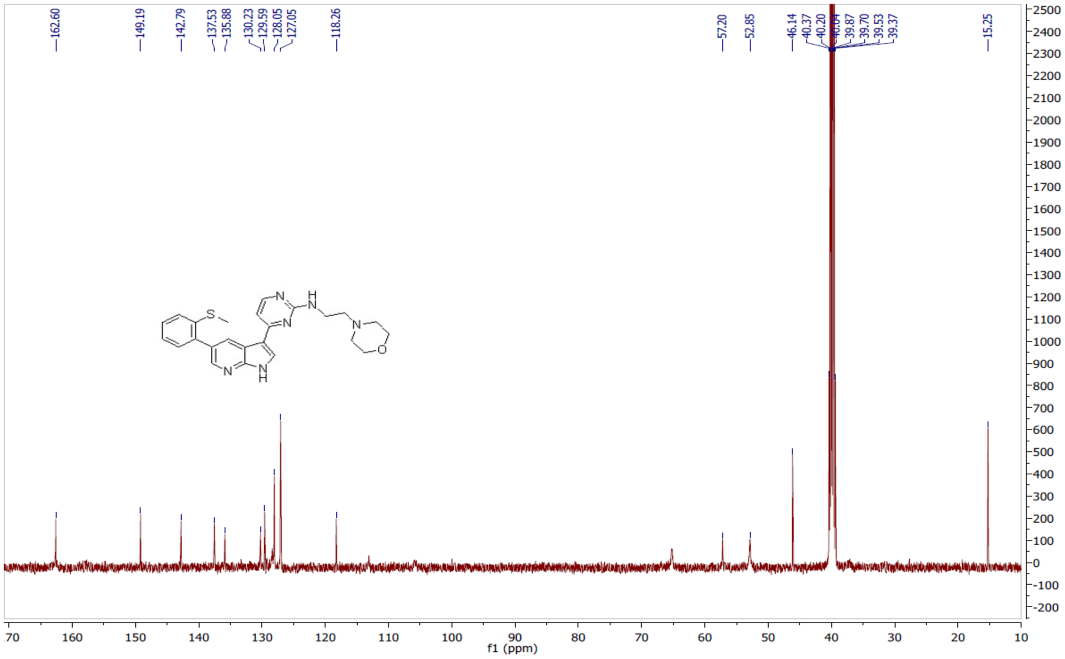

HRMS of US-748:

Qualitative Compound Report

**Data File**	US-845.d	**Sample Name**	US-845
**Sample Type**	Sample	**Position**	Vial 13
**Instrument Name**	Instrument 1	**User Name**	
**Acq Method**	vishal_12-01-13.m	**Acquired Time**	08-06-2016 PM 1:28:27
**IRM Calibration Status**	Success	**DA Method**	daily_report.m
**Comment**			

**Sample Group**		**Info.**
**Acquisition SW Version**	6200 series TOF/6500 series Q-TOF B.05.01 (B5125)	

Compound Table

Compound Label	RT	Mass	Formula	MFG Formula	MFG Diff (ppm)	DB Formula
Cpd 45: C24 H26 N6 O S	0.347	446.1895	C24 H26 N6 O S	C24 H26 N6 O S	-1.43	C24 H26 N6 O S

Compound Label	*m/z*	RT	Algorithm	Mass
Cpd 45: C24 H26 N6 O S	447.1967	0.347	Find by Molecular Feature	446.1895

MFE MS Spectrum

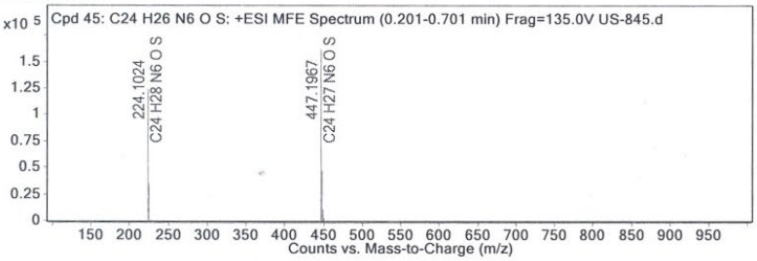

MS Spectrum Peak List

m/z	z	Abund	Formula	Ion
224.1024	2	124086.42	C24 H28 N6 O S	(M+2H)+2
224.6039	2	35365.42	C24 H28 N6 O S	(M+2H)+2
447.1967	1	161548.05	C24 H27 N6 O S	(M+H)+
448.1997	1	47511.23	C24 H27 N6 O S	(M+H)+
449.1983	1	11314.42	C24 H27 N6 O S	(M+H)+

Predicted Isotope Match Table

Isotope	*m/z*	Calc m/z	Diff (ppm)	Abund %	Calc Abund %	Abund Sum %	Calc Abund Sum %
1	224.1024	224.1017	-3.27	100	100	72.26	71.51
2	224.6039	224.6031	-3.51	28.5	29.3	20.59	20.95
3	225.1029	225.102	-4.13	8.29	8.82	5.99	6.31
4	225.6029	225.6023	-2.87	1.6	1.71	1.16	1.22

--- End Of Report ---

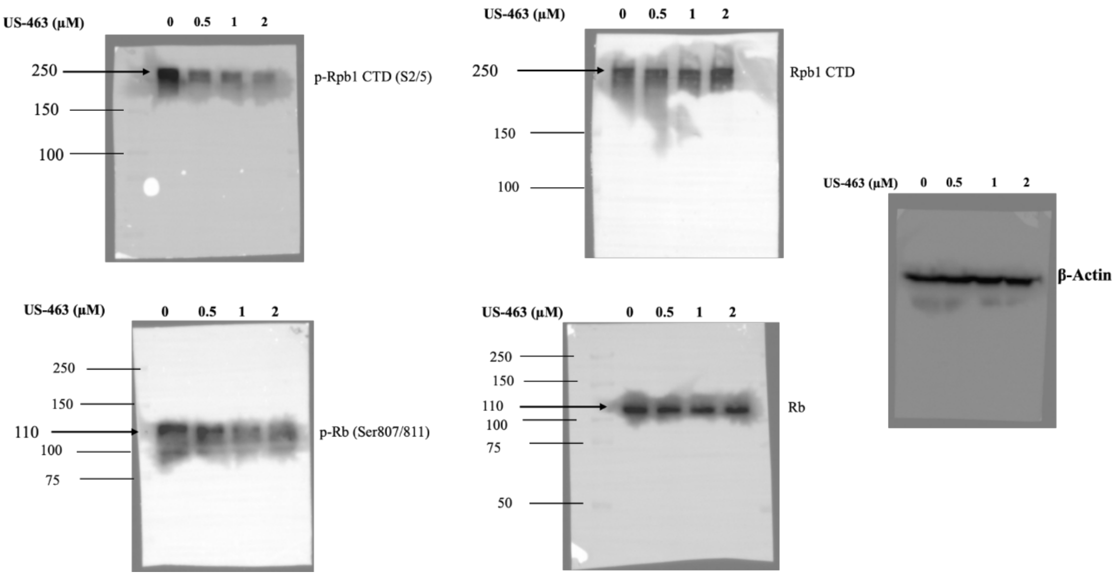

Original images of Western Blots related to Supplementary Figure S2.

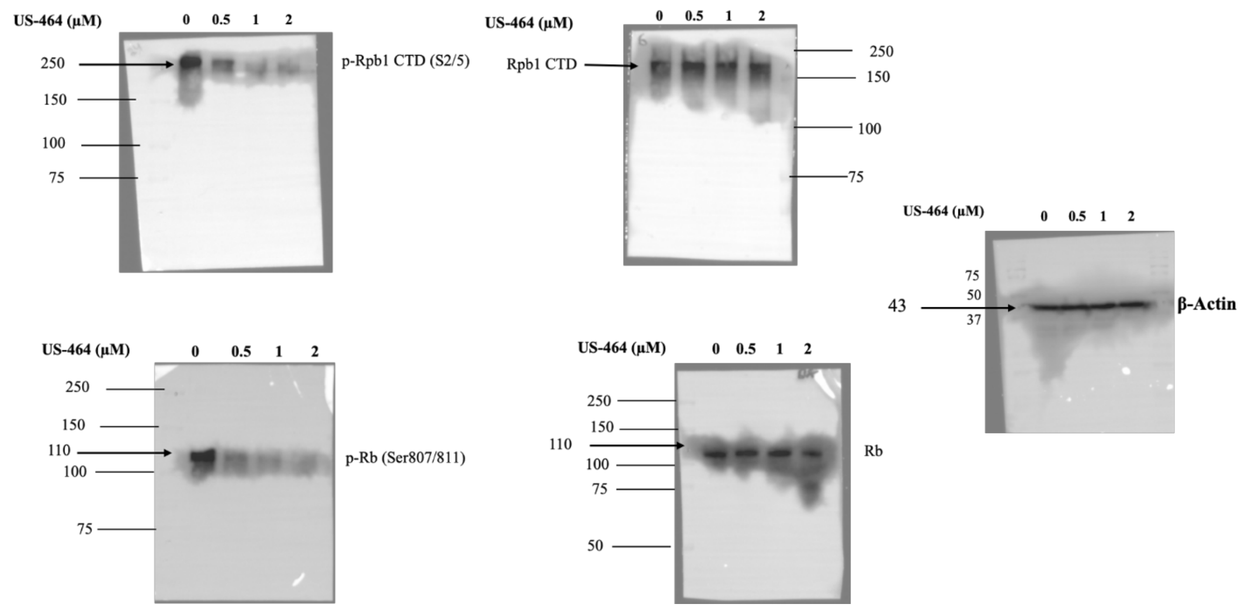

Original images of Western Blots related to Supplementary Figure S2.

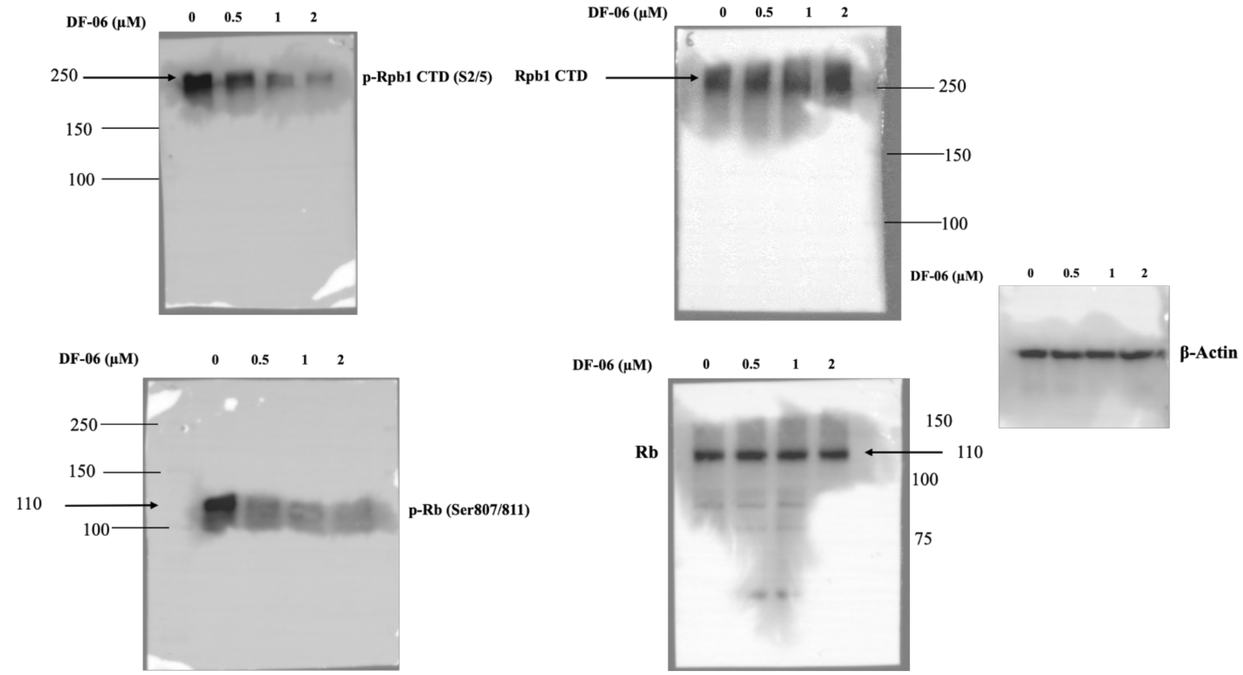

Original images of Western Blots related to Supplementary Figure S2.
